# Cryptosporidium Diarrhea in a Patient With Class IV Lupus Nephritis on Cyclophosphamide: An Underreported Case

**DOI:** 10.7759/cureus.89499

**Published:** 2025-08-06

**Authors:** Neha Sai P Doddapaneni, Saishirini Yerremreddy, Srinivas Nalabothula

**Affiliations:** 1 General Medicine, NRI Academy of Sciences, Guntur, IND; 2 General Medicine, Dr. Pinnamaneni Siddhartha Institute of Medical Sciences and Research Foundation, Vijayawada, IND; 3 Medicine and Surgery, Dr. Pinnamaneni Siddhartha Institute of Medical Sciences and Research Foundation, Vijayawada, IND

**Keywords:** acute parotitis, cryptosporidium infection, cryptosporidium spp, lupus nephritis, sle and lupus nephritis

## Abstract

*Cryptosporidium* is a protozoan parasite that typically causes self-limited diarrhea in healthy individuals but can result in prolonged, severe illness in those who are immunocompromised. While this infection is well recognized in HIV-positive patients, it is less frequently reported in individuals with lupus nephritis on immunosuppressive therapy. We report the case of a 27-year-old man with biopsy-confirmed class IV lupus nephritis who was receiving cyclophosphamide and corticosteroids. He was admitted with generalized edema, ascites, and declining kidney function. On hospital day 6, he developed acute-onset watery diarrhea. Stool microscopy identified *Cryptosporidium parvum*, and he was treated with nitazoxanide and rifaximin, resulting in improvement of his diarrhea. His hospital stay was complicated by the development of left-sided parotitis with abscess formation, bilateral pleural effusions, worsening renal failure requiring hemodialysis, and interstitial lung changes. He was discharged with symptomatic improvement but was later readmitted elsewhere, received additional cyclophosphamide, and ultimately died from septic shock and multi-organ failure. This case highlights the need for heightened clinical suspicion for opportunistic infections, such as cryptosporidiosis, in patients with lupus nephritis who present with new-onset diarrhea, particularly when on potent immunosuppression. It also reflects the broader challenges in managing such patients, where balancing immunosuppressive treatment against infection risk is both complex and critical.

## Introduction

Lupus nephritis (LN) is a serious and common manifestation of systemic lupus erythematosus (SLE), resulting from glomerular, tubulointerstitial, and vascular involvement that can lead to progressive renal impairment. It occurs in approximately 40% of patients with SLE, typically within the first five years of diagnosis. Gastrointestinal symptoms such as diarrhea, abdominal pain, nausea, vomiting, and anorexia are frequently reported in patients with LN [1]. Gastric involvement in SLE is also common, with gastritis and peptic ulcer disease being the most frequently observed pathologies, often exacerbated by the use of non-steroidal anti-inflammatory drugs (NSAIDs) and corticosteroids, which are routinely prescribed to manage lupus flares [2].

Diarrhea in patients with LN can result from a variety of causes, including medications such as NSAIDs, cyclophosphamide, and corticosteroids [3]. Among disease-related causes, lupus mesenteric vasculitis is the most common, followed by protein-losing enteropathy, intestinal pseudo-obstruction, acute pancreatitis, and rarer conditions such as celiac disease and inflammatory bowel disease [4].

Cryptosporidiosis, an infection caused by *Cryptosporidium* species, is transmitted via the fecal-oral route and is known to cause moderate to severe diarrhea. While it typically affects children and the elderly, individuals with acquired or congenital immunodeficiency are at significantly higher risk. Diagnosis is made through stool examination using special stains (e.g., modified acid-fast), antigen detection assays, or polymerase chain reaction (PCR) testing. In immunocompetent individuals, nitazoxanide is the FDA-approved treatment of choice. However, in immunocompromised patients, including those receiving immunosuppressive therapy, treatment is often less effective without immune reconstitution [5,6].

Although cryptosporidiosis has been well-documented in patients with HIV/AIDS, it is infrequently reported in individuals with SLE, making this a rare and noteworthy case.

## Case presentation

A 27-year-old male presented with progressive bilateral lower limb swelling for 20 days and abdominal distension for 15 days. He had a known history of hypertension, managed with Nebivolol, Chlorthalidone, and Benidipine for the past eight months. The pedal edema began as grade 1 but progressed rapidly to grade 4 within 20 days. Additionally, he reported dyspnea for 10 days, insidious in onset, non-progressive, and grade 3 in severity. It was not associated with orthopnea or paroxysmal nocturnal dyspnea. He also had a dry nocturnal cough but denied fever, jaundice, palpitations, abdominal pain, or decreased urine output.

Two months prior, the patient was diagnosed with class IV LN based on a renal biopsy performed for pedal edema and renal dysfunction. He was started on monthly cyclophosphamide (1 g/month) as per the NIH protocol, with the second dose administered three weeks before admission. The patient was also on prednisolone 40 mg (oral) and a tab of hydroxychloroquine 200 mg (oral) for lupus. He was also diagnosed with chronic kidney disease (CKD) with renal osteodystrophy and had been receiving teriparatide injections for generalized bone pain. His surgical history included right double-J stenting for right-sided pyelonephritis six months prior and right-sided pleural fluid aspiration for transudative effusion two months earlier, followed by video-assisted thoracoscopic surgery (VATS).

On examination, the patient appeared pale and had grade 4 pitting edema in both lower limbs. There was no clubbing, cyanosis, icterus, or lymphadenopathy. Respiratory examination revealed absent breath sounds in the left infra-scapular and infra-axillary areas. Abdominal examination showed non-tender distension with shifting dullness, suggestive of ascites.

Initial investigations revealed progressively elevated serum creatinine, increasing from 1.4 mg/dL eight months prior to 3.5 mg/dL two months earlier and reaching 4.1 mg/dL at presentation; hyperkalemia (5.6 mmol/L); hypoalbuminemia (2.2 g/dL); proteinuria (3+) on urine dipstick with corresponding 24-hour urine protein (4.5 g/day); low complement C3 (74.04 mg/dL); and normal ejection fraction (60%) on 2D echocardiography. The following lab results were obtained on further examination (Table 1).

**Table 1:** Lab results showing low complement C3 level and elevated serum creatinine, potassium, and phosphorus levels with proteinuria present. WBC: White Blood Cells; ESR: Erythrocyte Sedimentation Rate; A/G Ratio: Albumin to Globulin Ratio; RBC: Red Blood Cells

Parameter	Lab Value	Normal Value
Hemoglobin	10.2 g/dL	13–17 g/dL
Total WBC	12,000/cumm	4,000–10,000/cumm
Platelets	214,000/dL	150,000–450,000/dL
ESR	30 mm/hr	0–15 mm/hr
Creatinine	4.1 mg/dL	0.52–1.04 mg/dL
Serum Sodium	140 mmol/L	137–145 mmol/L
Serum Potassium	5.6 mmol/L	3.5–5.1 mmol/L
Serum Chloride	107 mmol/L	95–105 mmol/L
Serum Albumin	2.2 g/dL	3.4–5.4 g/dL
Serum Globulin	2.3 g/dL	2.0–3.5 g/dL
A/G Ratio	0.9:1	1–2
Urine Protein	3+	Nil
Urine RBC	1/hpf	Nil
Urine WBC	1/hpf	Nil
Complement C3	74.04 mg/dL	80–165 mg/dL
Complement C4	33.99 mg/dL	15–45 mg/dL
Phosphorus	10 mg/dL	0.8–1.5 mmol/L
Calcium	8.4 mg/dL	8.5–10.2 mg/dL
Serum Procalcitonin	2.66 ng/mL	< 0.5 ng/mL

Ultrasonography revealed bilaterally enlarged kidneys with altered echotexture, a right renal calculus, moderate ascites, and bilateral pleural effusion, more pronounced on the left. A repeat renal biopsy was performed on day 3 of admission due to worsening renal function. On day 4, the patient developed a moderate-grade fever without chills. Empirical cefoperazone-sulbactam was initiated, and blood/urine cultures were sent.

By day 6, he developed watery diarrhea (five to six episodes/day, non-bloody, non-mucoid) and was empirically started on albendazole. Fever subsided within 48 hours, and oral prednisolone was tapered from 40 mg to 30 mg. However, due to persistent volume overload, hemodialysis was initiated.

**Figure 1:**
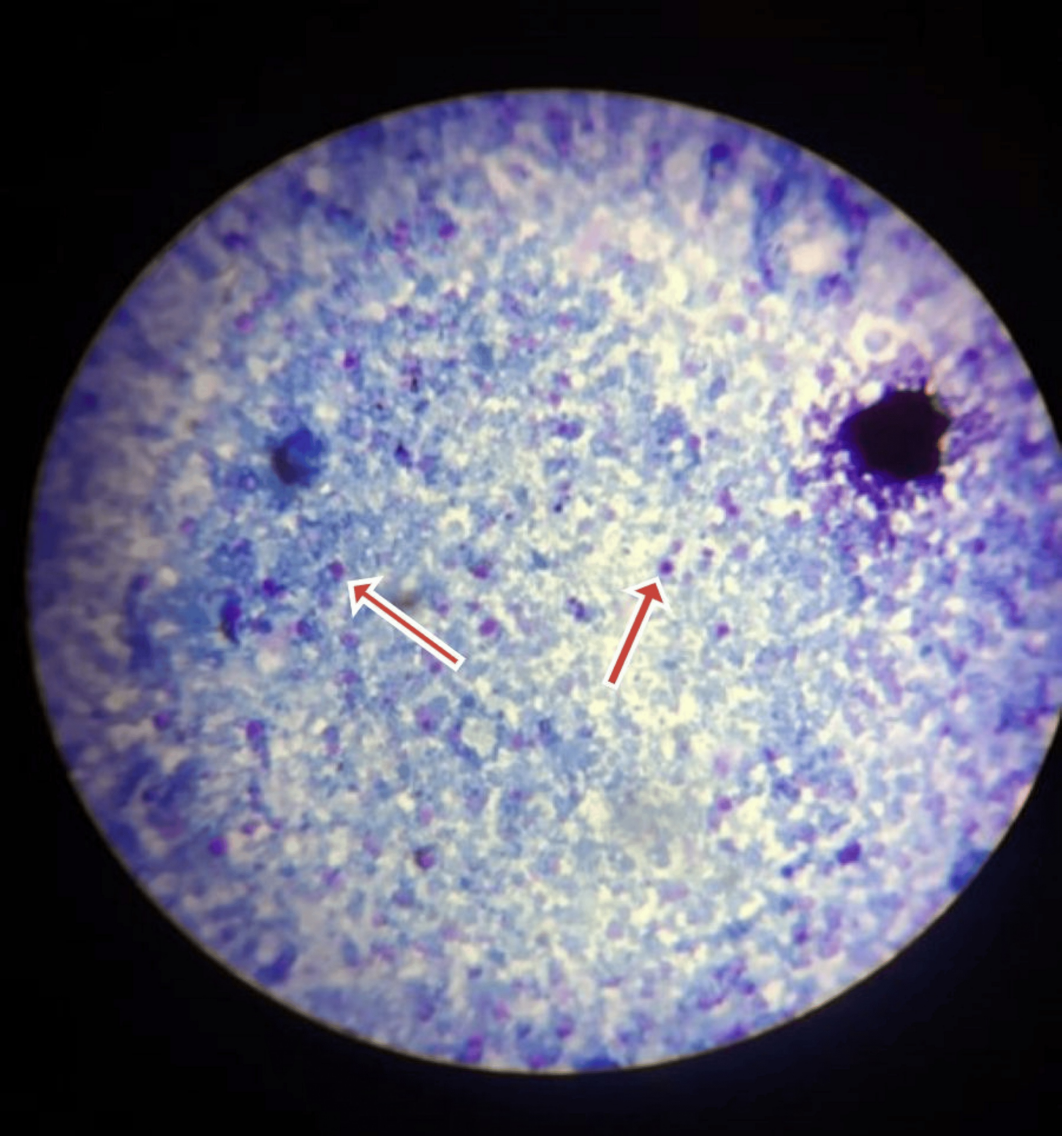
Cryptosporidium mature oocysts from a stool sample stained reddish purple (examined at 200–400 DPI) and appeared as spherical structures, measuring approximately 2–6 µm in diameter.

**Figure 2:**
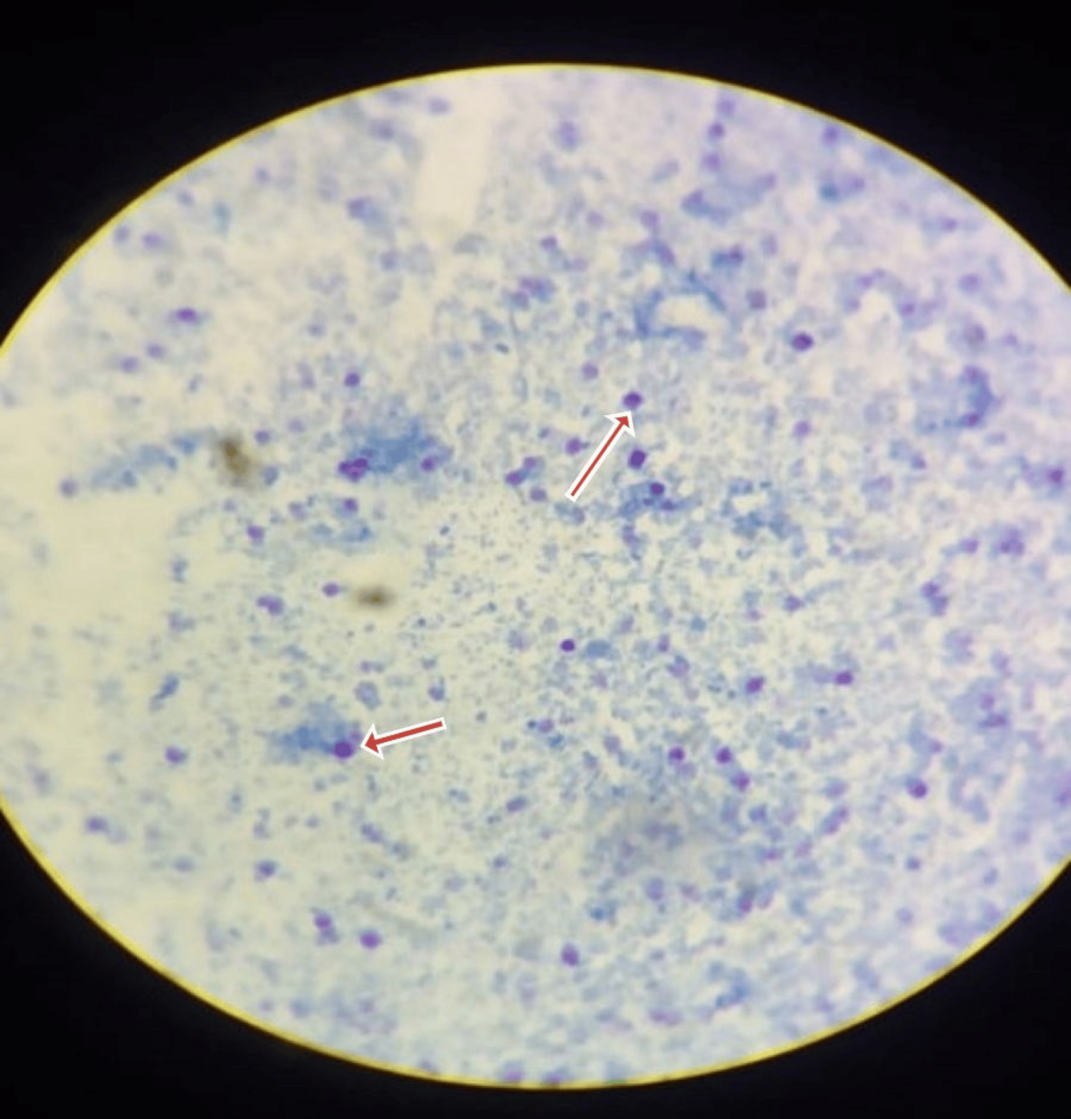
A modified acid-fast stain showing Cryptosporidium oocysts from a stool sample stained reddish purple at 200–400 DPI on microscopy.

**Figure 3:**
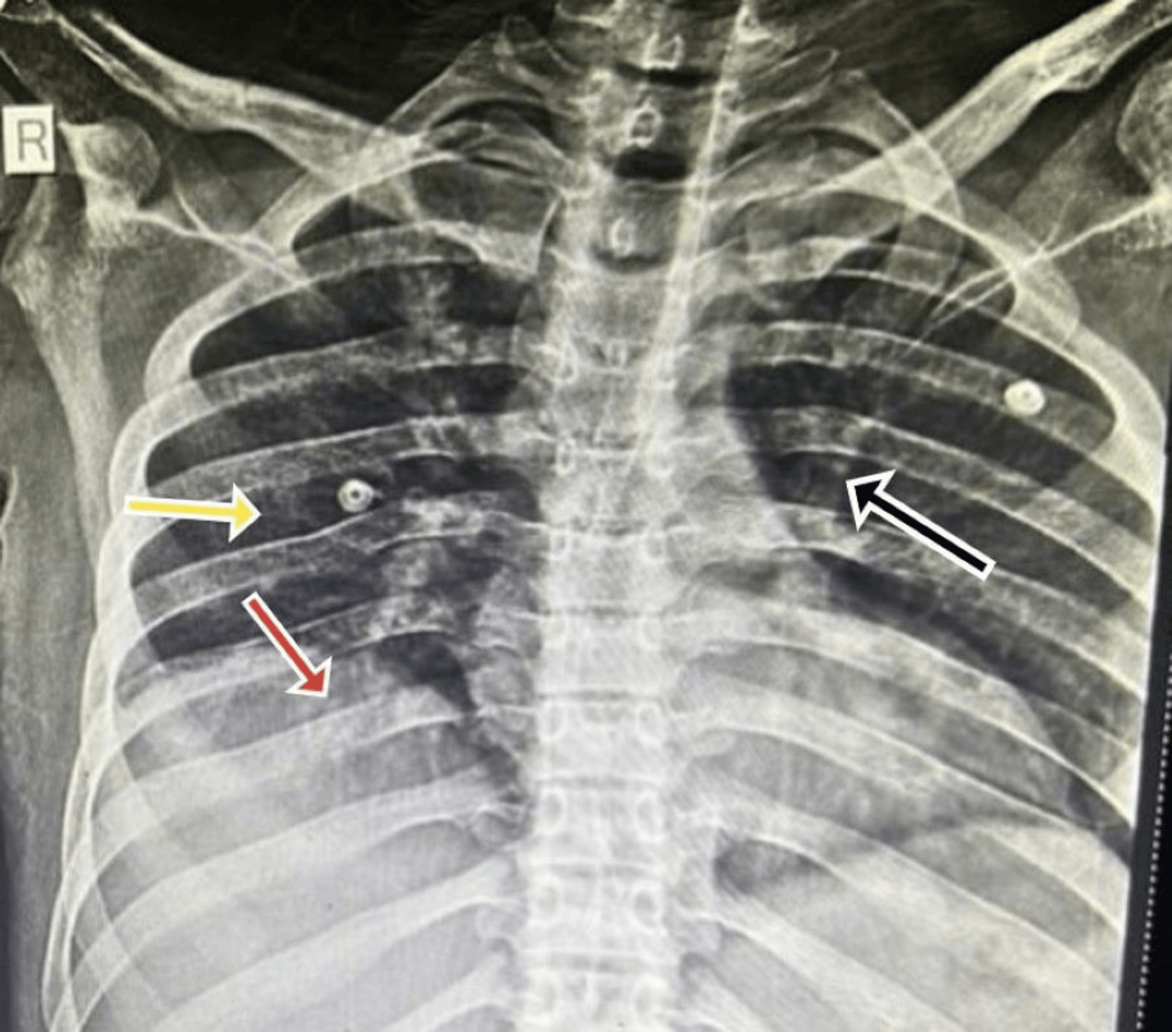
Chest X-ray showing bilateral interstitial infiltrates (black arrow), blunting of the Costo-phrenic angle indicating pleural effusion (red arrow), and pulmonary fibrosis (yellow arrow).

**Figure 4:**
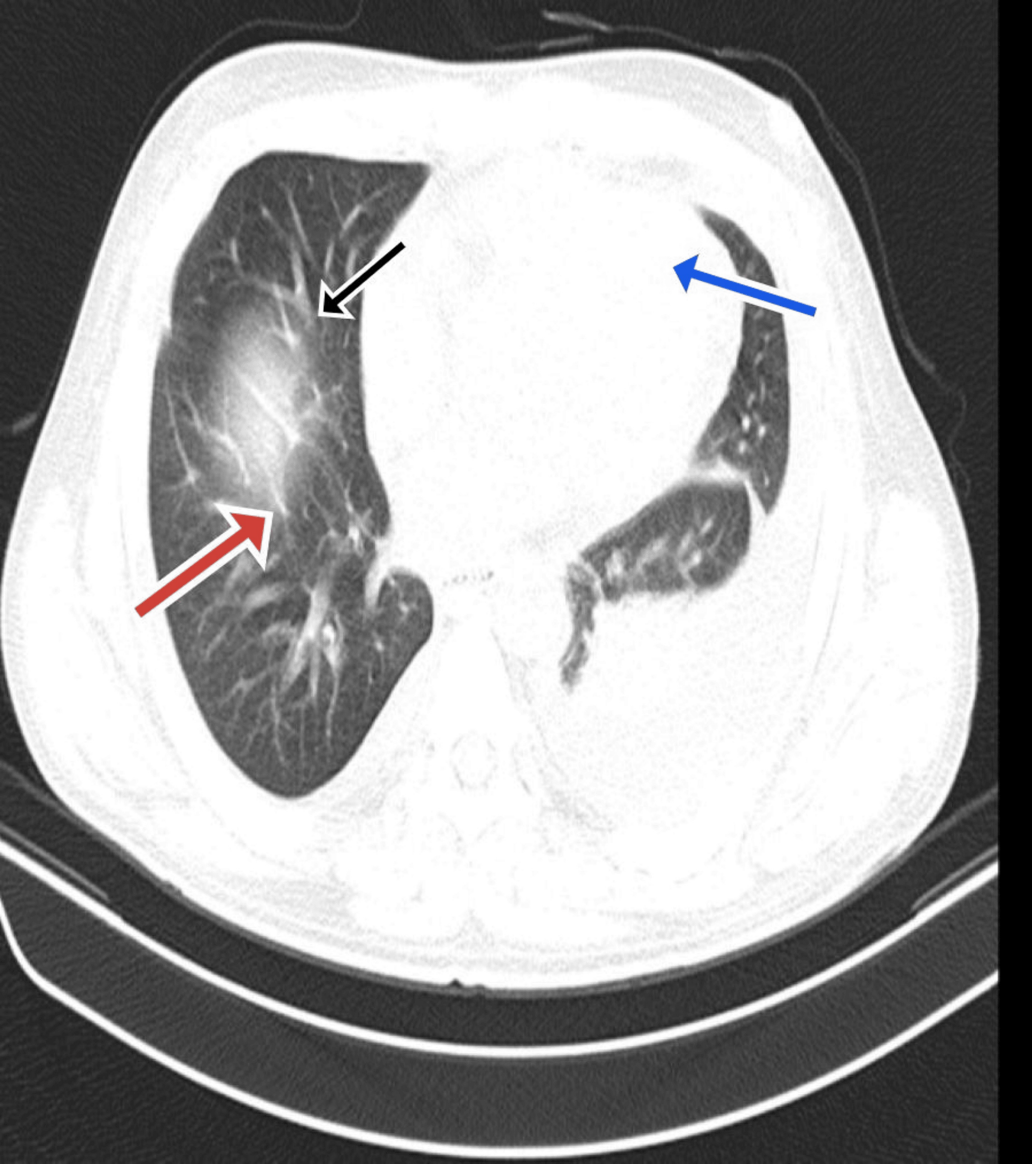
Plain CT chest showing ground-glass opacities (red arrow), pleural effusion (blue arrow), and interlobular septum thickening (black arrow).

Blood and urine cultures were sterile. Stool analysis confirmed the presence of *Cryptosporidium parvum *(Figures 1, 2). He was started on nitazoxanide 500 mg orally twice daily and rifaximin 550 mg orally twice daily. On day 10, the patient developed a productive cough; investigations, including a chest X-ray (Figure 3), chest CT (Figure 4), sputum culture, and the cartridge-based nucleic acid amplification test (CBNAAT), were ordered. Azithromycin (500 mg/OD) was added empirically. All microbiological investigations, including CBNAAT and sputum cultures, were negative.

**Figure 5:**
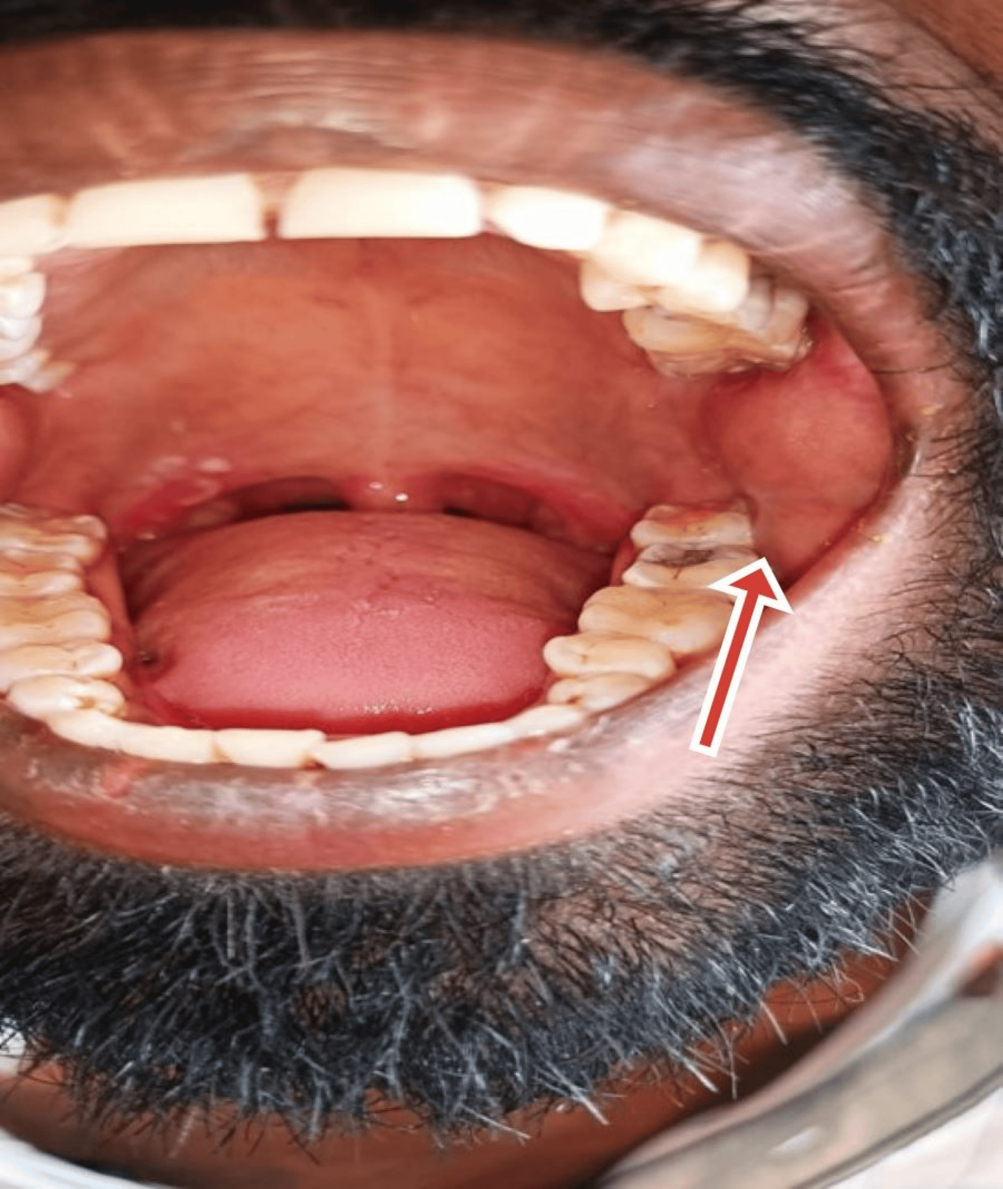
Swelling inside the mouth on the left side, where parotid gland swelling is present.

On day 11, the patient developed left cheek swelling that was warm and tender without lymphadenopathy (Figure 5). An ENT consultation revealed grade 2 tonsillar hypertrophy. Ultrasound of the cheek showed acute parotitis, a small evolving abscess, left submandibular gland enlargement, and a few sub-centimeter level II/III cervical nodes. Treatment was initiated with Amoxiclav and Chymoral Forte.

Over the next several days, the patient's parotid swelling reduced, fever subsided, and diarrhea and edema improved. However, he remained dialysis-dependent due to worsening renal function. He was discharged after three weeks with a plan for maintenance hemodialysis and tapering of immunosuppressants.

Unfortunately, the patient was lost to follow-up, later receiving another dose of cyclophosphamide at a different facility. According to the family, he subsequently developed septic shock and multi-organ dysfunction, ultimately resulting in his demise five days later.

## Discussion

Patients with SLE, especially those with LN, are at increased risk for infections due to the underlying immune dysregulation and use of immunosuppressive therapies. Cyclophosphamide, a potent cytotoxic agent used in the treatment of class IV LN, significantly increases this risk, particularly for opportunistic infections. While gastrointestinal (GI) symptoms are not uncommon in SLE, distinguishing between disease-related pathology and infectious causes is critical, particularly in immunocompromised individuals [7,8].

Cryptosporidiosis, caused by *Cryptosporidium parvum*, is a protozoan infection primarily known for causing self-limited diarrhea in immunocompetent individuals. However, in patients with compromised immunity, including those with HIV/AIDS, organ transplant recipients, and those on chemotherapy or immunosuppressive therapy. The infection can become chronic, severe, and even life-threatening [7]. Notably, cryptosporidiosis is underreported in patients with SLE, with only a few cases documented in the literature. This underreporting is likely due to underrecognition, especially among patients receiving immunosuppressive therapy. This case illustrates the diagnostic and therapeutic complexity that arises when such an infection occurs in an LN patient receiving immunosuppression.

Our patient presented with generalized edema, ascites, and renal dysfunction consistent with active LN. His treatment regimen, including corticosteroids and cyclophosphamide, rendered him highly immunocompromised. In patients with LN, diarrhea may arise from multiple potential etiologies. Medication-induced GI side effects, particularly from NSAIDs, cyclophosphamide, and corticosteroids, are common contributors. Additionally, disease-related gastrointestinal involvement should be considered, with lupus mesenteric vasculitis representing the most frequent cause. Other possible mechanisms include protein-losing enteropathy, intestinal pseudo-obstruction, and acute pancreatitis. Less commonly, conditions such as celiac disease and inflammatory bowel disease may also present in this patient population [3,4]. The onset of watery diarrhea during hospitalization, coupled with the absence of typical inflammatory or ischemic GI signs, warranted evaluation for infectious causes. Diagnosis was confirmed by stool microscopy using modified acid-fast staining, which revealed characteristic oocysts of *Cryptosporidium parvum*.

The management of cryptosporidiosis in immunosuppressed patients is particularly challenging. Nitazoxanide remains the only FDA-approved treatment, but its efficacy is limited in the absence of immune reconstitution [9,10]. Adjunctive therapy with rifaximin was used in our case, consistent with off-label approaches described in the literature [9]. Although diarrhea improved with this regimen, the patient developed further complications, including bilateral pleural effusions, pulmonary fibrosis, acute parotitis with abscess formation, and refractory volume overload requiring hemodialysis.

This clinical trajectory highlights the multifactorial burden of infections and complications in lupus patients who receive immunosuppressive therapy [9-11]. The patient's re-administration of cyclophosphamide at another center, despite ongoing dialysis dependence and prior infections, may have further compromised his ability to mount an adequate immune response, precipitating septic shock and multi-organ failure. This ultimately resulted in a fatal outcome.

This case adds to the limited literature on cryptosporidiosis in LN and highlights the importance of considering parasitic infections in immunocompromised patients with new-onset diarrhea [10,11]. It also reflects on the need for stringent follow-up, patient education regarding infection risk, and careful reevaluation of immunosuppressive therapy after serious infections [11].

## Conclusions

This case underscores the importance of considering opportunistic infections, such as *Cryptosporidium parvum*, in patients with LN who are receiving immunosuppressive therapy. Although rare, cryptosporidiosis can lead to significant morbidity and mortality in immunocompromised individuals. Prompt diagnosis and targeted treatment are essential, but outcomes may remain poor without careful immune modulation. Clinicians must weigh the risks of continued immunosuppression in patients with active infections and ensure coordinated, multidisciplinary care. Finally, this case highlights the critical need for close follow-up and patient education to prevent adverse outcomes in high-risk populations.
